# SurprisalAnalysis: an open-source software for information-theoretic analysis of gene expression

**DOI:** 10.1093/bioadv/vbaf291

**Published:** 2025-11-18

**Authors:** Annice Najafi

**Affiliations:** Department of Industrial and Manufacturing Engineering, California State Polytechnic University Pomona, Pomona, CA 91768, United States

## Abstract

**Summary:**

SurprisalAnalysis is an open-source R package with an accompanying web-based application that utilizes Surprisal analysis to extract patterns of genes that tend to get up or down regulated as a result of a biological process. Surprisal analysis frames gene expression values in thermodynamic terms and identifies entropy-driven constraints and relevant gene weights that allow the decomposition of each gene’s expression into a baseline (maximal entropy) component and one or more constraint-driven components. These components correspond to distinct biological modules or processes whose coordinated up or down regulation underlies the observed system dynamics.

**Availability and implementation:**

SurprisalAnalysis is written in R and is freely available on GitHub (https://github.com/AnniceNajafi/SurprisalAnalysis). The package is distributed under a permissive license to promote scientific collaboration and reproducibility. A web-based application with a Graphical User Interface (GUI) is hosted on https://najafiannice.shinyapps.io/surprisal_analysis_app/.

## 1 Introduction

The advent of modern transcriptomics technologies such as RNA-seq (Wang *et al.* 2009) and microarray (Schena *et al.* 1995) allowed capturing genome-wide gene expression over different timepoints and conditions resulting in high-dimensional data. Although these data convey significant biological information, their high dimensionality and noise complicate further analysis (Quackenbush 2001). The significant variability of gene expression across cells and time creates a burden in the extraction of meaningful patterns from data. To combat this issue, researchers often attempt to reduce data complexity through dimensionality reduction such as Principal Component Analysis (PCA) (Jolliffe 2002) or clustering (Qi *et al.* 2020). Although methods such as PCA find major variance components, they overlook biologically relevant signals that may come from smaller subpopulations or involve moderate changes (Zadran *et al.* 2014, Stegle *et al.* 2015). This results in the underestimation of important regulatory patterns through the use of these standard methods. Consequently, there is a need for data reduction methodologies that capture relative changes as well as absolute expression levels and improve the interpretability of transcriptomics data beyond variance-driven methods. Surprisal analysis which was originally developed by Levine and Bernstein in the 1970s to describe non-equilibrium chemical systems (Levine and Bernstein 1974, Levine 2009) meets this need through framing the transcription levels in thermodynamic terms where genes reside in a maximal entropy state and deviations from that baseline which are driven by underlying biological constraints manifest as patterns or modules of genes that tend to get up or down regulated together. Previously, Remacle *et al.* adapted this framework to cancer transcriptomics (Remacle *et al.* 2010) and Zadran *et al.* utilized Surprisal analysis to understand a specific cancer-related dynamic process, the Epithelial Mesenchymal Transition (EMT) (Zadran *et al.* 2014). Despite the informative and useful nature of Surprisal analysis for revealing coordinated regulatory modules, currently, no tools exist for the accessible implementation of Surprisal analysis thereby creating a barrier to its broader adoption in transcriptomics workflows. Here, we present the first Surprisal analysis R package and accompanied web-based application to increase the accessibility of this powerful information-theoretic method to the scientific community. We provide detailed instructions to use the methods within the R package on gene expression data. We then apply it to two distinct datasets to demonstrate how Surprisal analysis can be used to reveal biologically relevant gene modules.

## 2 Software description

### 2.1 Surprisal analysis procedure

The SurprisalAnalysis R package is based on the same methodology described in the Supplementary material of Kravchenko-Balasha *et al.* (2012). The algorithm behind the R package operates through the following five steps:

1. First, the entries in the gene expression matrix (*X*) are transformed into a log scale. We denote the resulting log-transformed expression matrix as *Y*, where rows correspond to genes and columns to samples. To prevent errors during log transformation, a small pseudocount (ϵ=1e−6) is added to all data points ($Y_{i}(T)=\ln(X_{i}(T)+\epsilon )$). Alternatively, the R package or web-based application can instead use the log1p(X) transformation. We have further assessed the robustness of Surprisal analysis to the normalization and zero handling choices and present the results in later sections.
2. The connectivity matrix (which is a symmetric matrix) is found through the following equation where *T* and $T^{{}^{\prime }}$ are two different timepoints or samples (*T* and $T^{{}^{\prime }}$  ∈  {0,1,2,…,N}), *i* is a specific gene in the data and $C_{T,T^{{}^{\prime }}}$ is the connectivity matrix element corresponding to timepoints (or samples) *T* and $T^{{}^{\prime }}$:
  (1) $$C_{T,T^{{}^{\prime }}}=\sum_{i}Y_{i}(T)Y_{i}(T^{\prime })$$
3. *P* holding the eigenvectors of *C* and $\omega_{\alpha }^{2}$ corresponding to the eigenvalues ($\omega_{\alpha }$ is the αth singular value) are found through eigenvalue decomposition:
  (2) $$CP_{\alpha }=\omega_{\alpha }^{2}P_{\alpha },\alpha \in \{0,1,2,\ldots ,N\}$$
4. The constraints or Lagrange multipliers ($\lambda_{\alpha }$ where α corresponds to patterns of genes) can be found through the below equation:
  (3) $$\lambda_{\alpha }(T)=P_{T\alpha }\omega_{\alpha }$$
5. The weights of each gene (*i*) in each pattern or constraint ($\lambda_{\alpha }$), are found through the below equation and expressed in a vector $G_{\alpha }$ where the $G_{i\alpha }$ element is the weight of gene *i* in pattern $\lambda_{\alpha }$ (we note that $C=Y^{T}Y$):
  (4) $$G_{\alpha }=\omega_{\alpha }^{-1}YP_{\alpha }$$

The steps above can be used to reconstruct the equation below where $\ln X_{i}^{0}$ denotes the steady-state expression level of gene *i* (we write $\ln X_{i}(T)$ as shorthand for $\ln(X_{i}(T)+\epsilon )$):


(5) $$\ln(X_{i}(T))=\ln(X_{i}^{0})+\sum_{\alpha \geq 1}G_{i\alpha }\lambda_{\alpha }(T)$$

Noting that:


(6) $$\ln(X_{i}^{0})=G_{i0}\lambda_{0}$$

Here, $\lambda_{0}$ represents the balance or steady state of the system, and captures the baseline transcriptional program. Genes with the largest weights in $G_{0}$ are those whose expression is least affected by the perturbations or processes driving deviations in higher-order patterns. All observed changes in gene expression relative to this baseline state are interpreted as constraint-driven deviations described by the higher-order $\lambda_{\alpha }$ terms. We note that the balance or steady state of the system remains constant across samples. This constant component defines the unperturbed reference from which biologically meaningful variations emerge, while the higher-order constraints quantify coordinated shifts in expression driven by underlying regulatory processes.

We have provided a summary of all mathematical symbols and their definitions in Table 1.

**Table 1.** Notation used in surprisal analysis.

Symbol	Definition
*X*	Raw gene-expression matrix. The individual entries are $X_{i}(T)$; rows = genes (*i*), columns = samples (*T*)
*Y*	Log-transformed matrix, $Y_{i}(T)=\ln(X_{i}(T)+\epsilon )$ or $\text{log}\;1p(X_{i}(T))$.
ϵ	Small pseudocount (e.g. $10^{-6}$) to avoid undefined logs.
$T,T^{\prime }$	Sample indices, $T,T^{\prime }\in \{0,\ldots ,N\}$.
*N*	Number of samples - 1 (sample indices start from 0).
*i*	Gene index.
*C*	Connectivity matrix, $C=Y^{⊤}Y$ (symmetric (N+1)×(N+1), since sample index starts at 0).
$C_{TT^{\prime }}$	Entry of *C* between samples *T* and $T^{\prime }$, $C_{TT^{\prime }}=\sum_{i}Y_{i}(T)Y_{i}(T^{\prime })$.
$P_{\alpha }$	Eigenvector of *C* for pattern α.
$P_{T\alpha }$	Entry of *P* at sample *T*, pattern α.
$\omega_{\alpha }$	αth Singular value of *Y*.
$G_{\alpha }$	Gene-weight vector, $G_{\alpha }=\omega_{\alpha }^{-1}YP_{\alpha }$.
$G_{i\alpha }$	Weight of gene *i* in pattern α.
$\lambda_{\alpha }(T)$	Constraint (Lagrange multiplier) for pattern α in sample *T*.
$\lambda_{0}$	Balance (steady-state) constraint; largest $G_{i0}$ represents least perturbed genes.
$\ln(X_{i}^{0})$	Baseline log-expression of gene *i* in the balance state.

### 2.2 Installation

The package is currently accessible through CRAN or GitHub.

To install the R package through CRAN, simply run:


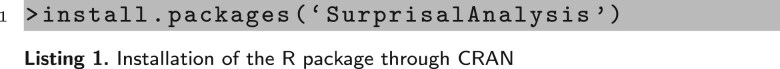


Alternatively, to install the R package through Github, please install devtools and run the code below:


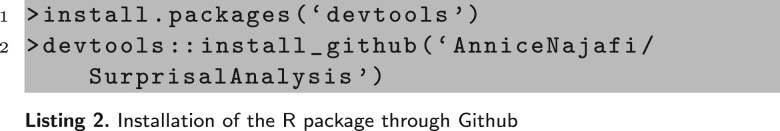


### 2.3 Input files

Both the SurprisalAnalysis R package and its companion web application accept a single Comma Separated Value (CSV) file containing a gene‐by‐sample expression matrix. The expected format is:

- **Header row:** The first row is supposed to hold sample names. The sample names can be a combination of numeric or character values and may represent timepoints or conditions.
- **First column:** Gene identifiers.
- **Data block:** Rows *i* and columns *j* contain the raw expression value $X_{ij}$ for gene *i* in timepoint/sample *j*.

The package will automatically detect and parse this layout; no additional files are required.

### 2.4 Apply surprisal analysis

To read the input file and run Surprisal analysis, the following code can be utilized:


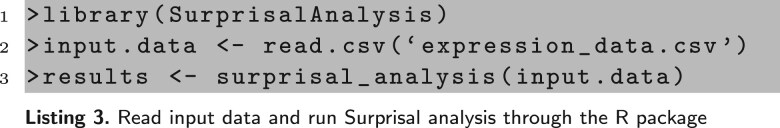


### 2.5 Gene ontology (GO) enrichment analysis

To understand which biological processes correspond the most or least to each pattern, genes with the highest or lowest weights can be extracted from the data and explored using GO enrichment analysis. GO enrichment analysis is performed on the subset of genes that are most influential within a given pattern. The influence of a gene is quantified by its weight in defining the corresponding constraint ($G_{ij}$ is the weight of gene *i* in pattern *j*). To extract the relevant set of genes, the user specifies a percentile cutoff, to include only genes above that specific percentile in the analysis. Input datasets may contain gene identifiers in various formats. By default, the package assumes gene identifiers are in the form of gene symbols, but this can be modified using the key_type argument to match any of the supported identifier types for *Homo sapiens* or *Mus musculus*. The complete list of supported key types can be obtained in R by running:


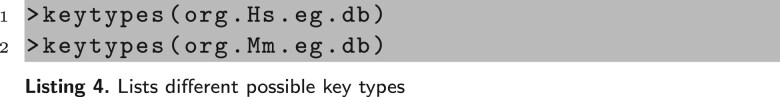


The R package and the web-based application both utilize the enrichGO function from the clusterProfiler package to perform GO enrichment analysis (Wu *et al.* 2021). By default, clusterProfiler (Wu *et al.* 2021) applies the Benjamini–Hochberg (BH) procedure for multiple testing correction. Users can modify this behavior by setting the pAdjustMethod argument to one of the following options: “bonferroni” (Bonferroni), “holm” (Holm), “hochberg” (Hochberg), “hommel” (Hommel), “BH” (Benjamini–Hochberg), “BY” (Benjamini–Yekutieli), or “none”. These options are consistent with those available in the enrichGO function of clusterProfiler for ease of usability.

The enrichGO function also requires the user to specify which branch of the Gene Ontology should be tested. Available options are: “BP”, “MF”, and “CC”. “BP” (Biological Process) corresponds to pathways and higher-level cellular programs such as cell cycle progression, apoptotic processes, or immune responses. “MF” (Molecular Function) corresponds to the basic biochemical activities of gene products, such as ATP binding, protein kinase activity, or DNA helicase activity. Lastly, “CC” (Cellular Component) corresponds to the subcellular location or macromolecular complex where gene products act, such as mitochondrial matrix, ribosome, or plasma membrane.

By default, the application and package use “BP”. However, users may select “MF” or “CC” depending on the biological question of interest. These options correspond directly to those supported by clusterProfiler (Wu *et al.* 2021). The GO analysis will consider all annotated genes within the selected organism database as the background or universe.

Because the enrichGO function in the clusterProfiler package (Wu *et al.* 2021) requires input as a vector of Entrez gene IDs, the SurprisalAnalysis package automatically converts user-supplied gene identifiers into Entrez IDs using the AnnotationDbi package (Pagès *et al.* 2023).

The flip argument is a logical flag. If TRUE, the function flips the orientation of each pattern by multiplying the corresponding constraint values ($\lambda_{\alpha }(T)$) by −1.

Below, we provide an example where genes in the 95th percentile and above of the first Lagrange multiplier are extracted and GO enrichment analysis using Homo Sapiens genes with default settings is applied on them.


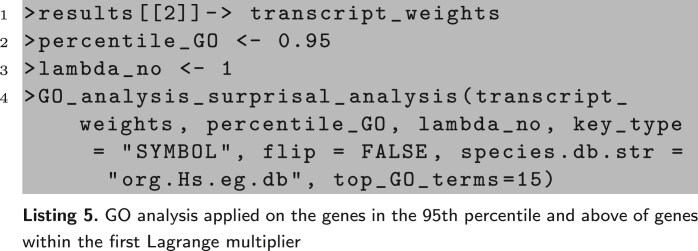


### 2.6 Graphical user interface (GUI)

The SurprisalAnalysis R package is accompanied by a web-based application, which can be either accessed directly through this link: https://najafiannice.shinyapps.io/surprisal_analysis_app/ or loaded through the package using the command provided below.


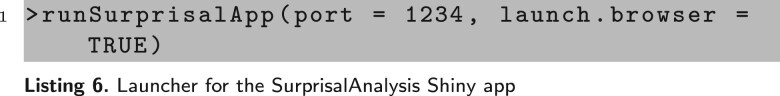


## 3 Preprocessing analysis

To understand the differences between the two zero handling methods of log1p(X) and pseudocount addition, we tested both of them on an RNA sequencing dataset (Fu *et al.* 2015) with raw RNA sequencing read counts. This dataset contains gene expression data extracted from luminal and basal cells of mice mammary glands from 2 day lactating and 18.5 day pregnant mice.

**Figure 1.**
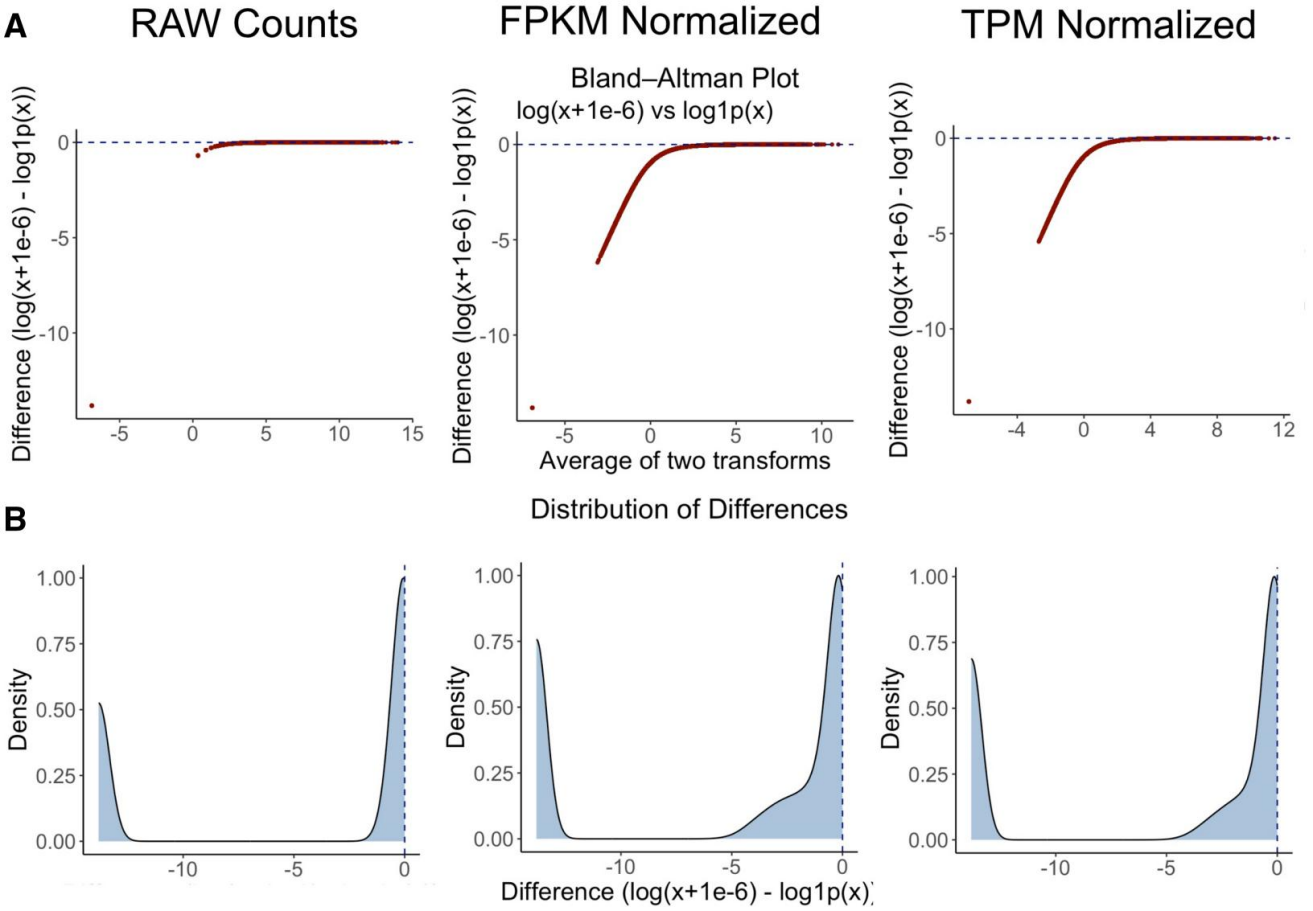
Three different normalization methods applied to the Fu *et al.* (2015) dataset. Panel A shows the Bland–Altman plots for raw counts, FPKM normalized, and TPM normalized data from left to right, respectively. Panel B shows the distribution of differences between the values using the two different zero handling methods.

We performed FPKM and TPM normalizations (Zhao *et al.* 2021) on the data (Fu *et al.* 2015) and repeated the analysis. To visualize the differences between the two zero handling methods, we have shown the corresponding Bland–Altman curves and their distributions in Fig. 1A. As shown in the figure, the differences between the two methods were less pronounced for raw read counts compared to FPKM and TPM normalized data. In all cases, a significant proportion of the data showed values clustered near zero (Fig. 1B), indicating that a significant portion of the genes are minimally affected by the choice of transformation. However, there is still a clustering of values with substantial deviations which reflects the sensitivity of low-abundance genes to the particular zero handling method applied.

**Figure 2.**
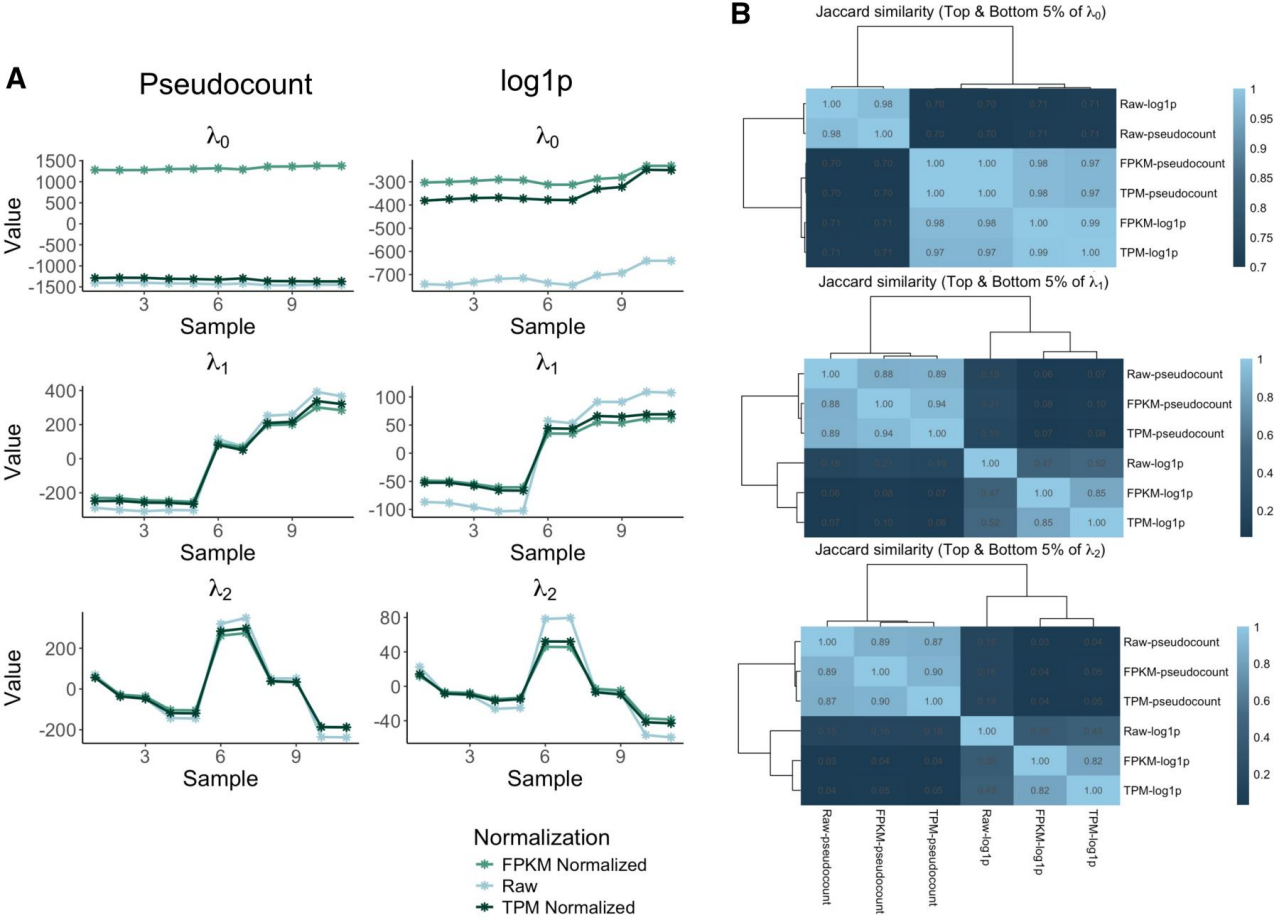
Effect of normalization and zero-handling on Surprisal analysis results. (A) Temporal dynamics of the first three constraints ($\lambda_{0}$, $\lambda_{1}$, and $\lambda_{2}$) extracted using Surprisal analysis with either pseudocount (left) or log1p (right) transformations. Analyses were performed on raw counts, FPKM-normalized, and TPM-normalized data. Despite differences in scaling, the overall dynamics of constraints are highly similar across normalization methods. (B) Jaccard similarity heatmaps comparing overlap of the top and bottom 5% of genes by gene weights across different normalization and zero-handling methods for $\lambda_{0}$, $\lambda_{1}$, and $\lambda_{2}$ from top to bottom, respectively. High similarity is observed between normalization methods within the same zero-handling approach, while greater variability arises from the choice of zero-handling method.

To understand the effects of normalization and zero handling methods on the final results, we continued by applying Surprisal analysis to all cases and have shown the time dynamics of the first three constraints of each normalization case with the two different zero handling methods in Fig. 2. As shown in Fig. 2A both the pseudocount and the *log*1*p* methods extracted similar pattern dynamics from the data where the change in sign of the first and second patterns happen at approximately the same values regardless of the zero handling or normalization method. On the other hand, when we extracted the top and bottom 5% of genes in terms of gene weights in each pattern, and calculated the Jaccard similarity index (which measures the ratio of the intersection of two sets to their union), we observed an agreement between the genes extracted using any normalization method but a lack of agreement between the genes extracted using the two different zero handling methods except for the baseline state ($\lambda_{0}$). Overall, the results suggest a lack of dependence of Surprisal analysis results to the particular normalization method used. This suggests that normalization is not necessarily required for the application of Surprisal analysis.

## 4 Applications and findings

**Figure 3.**
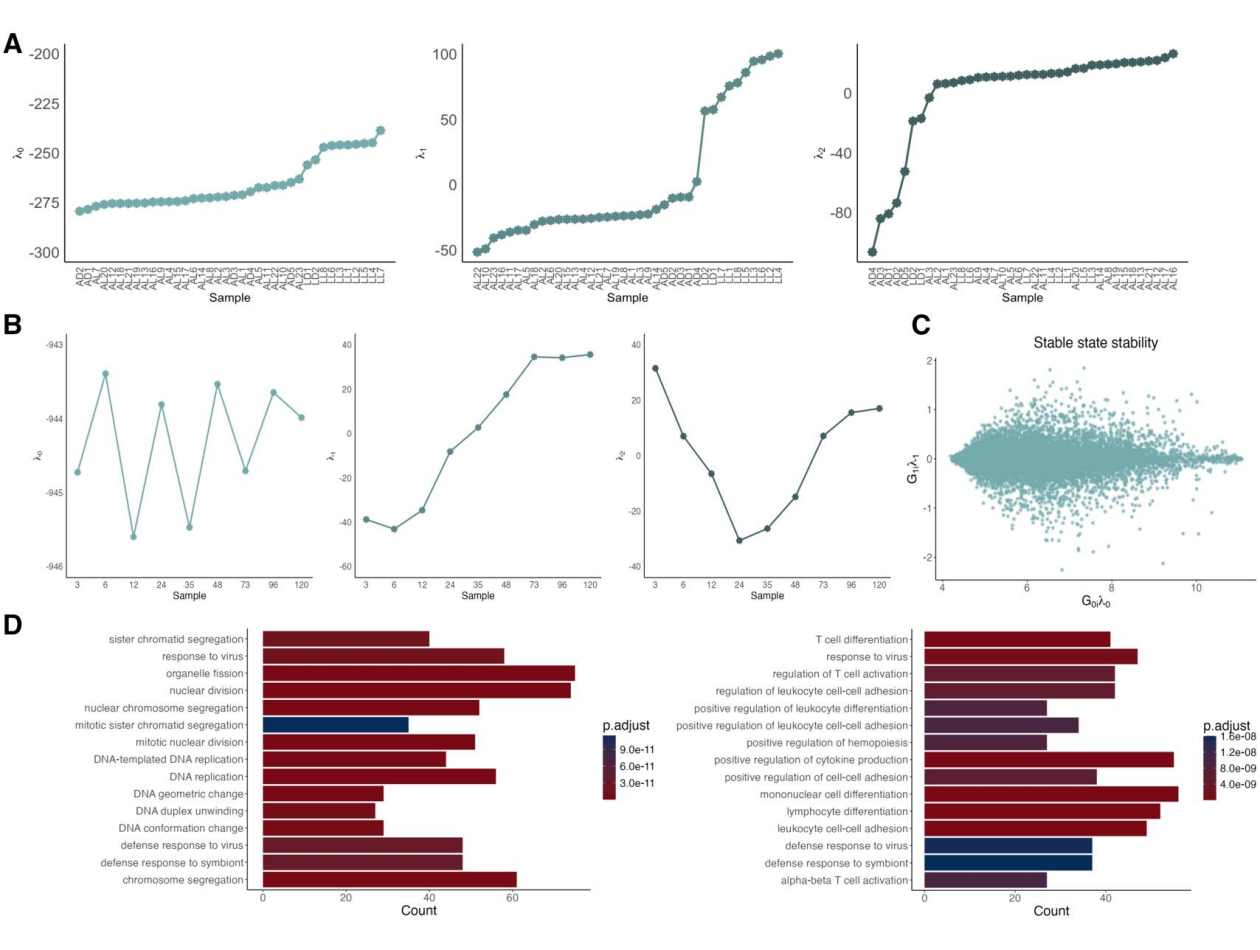
Surprisal analysis applied to two distinct datasets using the SurprisalAnalysis package. (A) The application of Surprisal analysis on the Bogaert *et al.* (2018) data. (B) The temporal evolution of the Lagrange multiplier values for the balance state and two constraints of the Burt *et al.* data for one of the Th0 phenotype replicates (Burt *et al.* 2022). (C) The relationship between the weight of genes in $\lambda_{1}$ and $\lambda_{0}$. The plot confirms that genes that play an important role in $\lambda_{0}$ (meaning they contribute to the steady state) have a weight close to 0 in the $\lambda_{1}$ pattern. (D) The GO enrichment analysis applied to the $\lambda_{1}$ and $\lambda_{2}$ patterns from the same data from Burt *et al.* (2022), respectively.

To test the functionality and application of our package, we applied it to two sets of gene expression data. First, we applied Surprisal analysis using our R package to the FPKM normalized gene expression data provided in supplementary material Table S5 of Bogaert *et al.* (2018) (Table S5 refers to the supplementary material of Bogaert *et al.* and not this manuscript). In Fig. 3A, we show the user interface of the software and demonstrate that the extracted constraint values from the SurprisalAnalysis package match the results of Bogaert *et al.* (2018).

Next, we used the SurprisalAnalysis package to analyze time-course data of T helper cells undergoing in-vitro polarization (Burt *et al.* 2022). For demonstration purposes, we have included the application of Surprisal analysis using the R package on the Th0 dataset (Burt *et al.* 2022) in Fig. 3B. The results indicated that the first Lagrange multiplier values changed signs around the 35 hour timepoint and GO enrichment analysis on the genes with the highest weights in this pattern indicated an association of this constraint with cell cycle related changes. On the other hand, a similar approach on the second constraint indicated an association with T-cell differentiation (3B-D). We note that these findings are consistent with previous reports of the emergence of proliferating T-cells and cytokine expressing cells approximately at the timepoints of sign changes of the relevant constraint (Proserpio *et al.* 2016). This indicates that the SurprisalAnalysis package provides a straightforward implementation of a powerful method for identifying important biological processes from gene expression data.

## 5 Runtime and peak RAM usage

**Figure 4.**
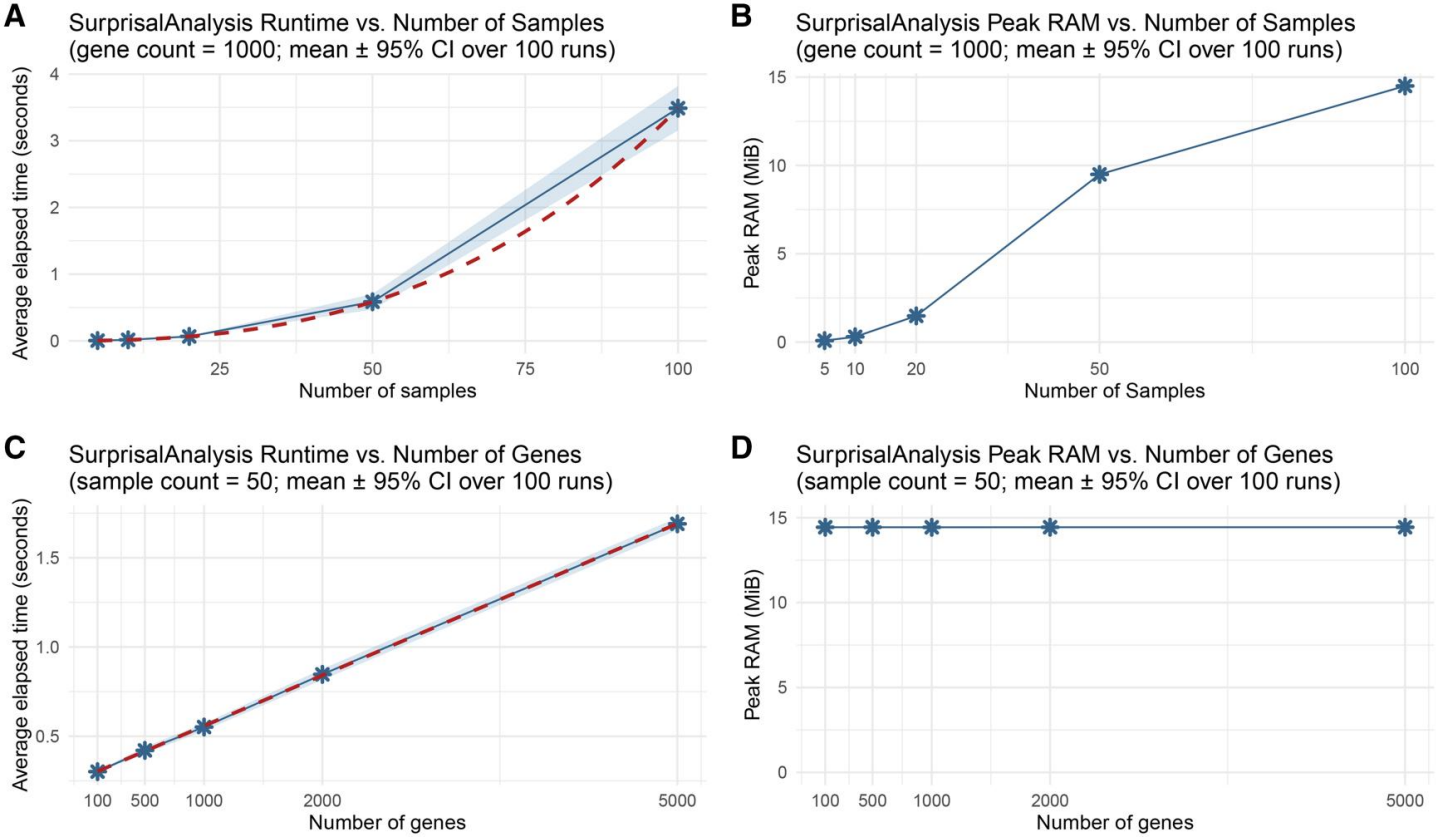
Runtime and peak RAM usage recorded and visualized over different gene and sample sizes. (A and B) The average elapsed time and peak RAM usage as the number of samples increases from 5 to 100 over 100 runs with a constant gene count of 1000. (C and D) The average runtime and peak RAM usage for a constant sample size (50) and a range of gene numbers. Confidence intervals were calculated and shown as shaded regions. Peak RAM is calculated in units of Mebibyte (MiB). Because the variability across replicates is extremely small in panels B and D, the 95% confidence intervals collapse onto the mean line and are visually indistinguishable.


To understand how runtime scales in our SurprisalAnalysis package, we performed a sensitivity analysis on a normal laptop running on an Apple M1 Pro CPU with 16 GB memory. First, we generated random input matrices, varied the number of samples, ran Surprisal analysis, (while keeping the number of genes constant at 1000) and recorded the average elapsed time over 100 runs. As shown in Fig. 4A, runtime increased monotonically. We fitted a cubic polynomial to these runtimes and overlaid the resulting cubic curve in the same panel (Fig. 4A). The cubic polynomial curve was fitted to the data using orthogonal polynomials, as implemented in the following code, where *results_df* contains the recorded mean runtimes stored in a *mean_time* column and *n_samples* denotes the number of samples (The 95% confidence interval for the mean was calculated using a t-distribution, based on the mean and standard deviation obtained from repeated runs of the algorithm).

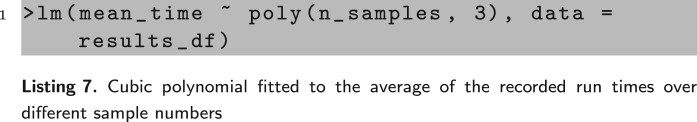


Next, we fixed the sample count at 50 and increased the number of genes; in that scenario, the elapsed time followed a linear trend (Fig. 4B). The shaded intervals shown in the plots were empirically estimated from the mean and standard deviation of the values across replicate runs which reflects the variability in repeated measurements.

We also recorded the peak Random Access Memory (RAM) usage using the peakRAM package in R (Quinn 2017). As shown in Fig. 4B peak RAM usage increases as the number of samples increases. However, the peak RAM usage remains constant as the number of genes increases (Fig. 4D). This is explained by the fact that the algorithm performs eigenvalue decomposition on the sample–sample connectivity matrix, so RAM usage scales with the number of samples rather than the number of features (genes).

## 6 Limitations

Although this methodology provides a robust method for the extraction of meaningful information from high-dimensional data, it is currently only applicable to bulk sequencing data and in cases of non-linear dynamics or where gene expression changes are influenced by drop-out effects which is a common occurrence in single-cell RNA sequencing, the current framework may require additional corrections. To address these concerns, DeMeo and Berger introduced a Surprisal Component Analysis (SCA) method to extend Surprisal-based dimensionality reduction to single-cell RNA sequencing data. SCA compares the expression of each transcript to its neighbors and calculates a “surprisal score” to quantify the more or less probable overexpression or underexpression of a gene or transcript (DeMeo and Berger 2023). However, because SCA is tailored specifically for single-cell data, it is not designed for bulk sequencing, and the only freely available implementation is in Python. Our future work will focus on extending the compatibility of these implementations with emerging single-cell modalities.

## Data Availability

The Burt *et al.* (2022), and Fu *et al.* (2015) datasets were obtained through GEO with accession codes GSE200250 and GSE60450, respectively. The FPKM normalized dataset from Bogaert *et al.* was downloaded from Table S5 of the Bogaert *et al.* (2018) research article.
